# L’intégration des services en santé : une approche populationnelle: Yves Couturier, Lucie Bonin and Louise Belzile, Montréal : Les Presses de l’Université de Montréal, 2016, pp 264, ISBN: 978-2-7606-3567-8 (paperback, available only in French)

**DOI:** 10.5334/ijic.4660

**Published:** 2019-01-25

**Authors:** Paul Wankah

**Affiliations:** 1 Université de Sherbrooke, Quebec, CA

**Keywords:** integrated care, older adults, population-based approach, case management

This book, authored by Quebec-based researchers Yves Couturier, Lucie Bonin and Louise Belzile, makes a strong and compelling case for the *population-based approach* as a fundamental principle of integrated health and social services. The central theme of this book emphasizes that designing, implementing, routinizing, evaluating and sustaining integrated services for individuals and the population necessitates the collective action of multiple stakeholders. This book is rich in tenets, theories and frameworks underpinning the foundational pillars of integrated care; for example, patient-centered care, care coordination, and patient engagement. The authors contribute to the understanding of integrated care by regularly linking its principles to practical applications for public policies, managerial/clinical practices and tools. In each chapter, the authors’ main ideas are summarised in information boxes, while clinical cases are used to delineate or discuss integrated care principles. This book consists of 11 chapters distributed in 2 sections.

The book begins with a brief introduction positioning integrated care as an innovative shift in the paradigm of addressing complex health and social needs of clients – from organising services based on the needs of the health system to organising services based on the needs of the client. The rationale for integrating health and social services is discussed, emphasizing the need to improve the satisfaction of clients, quality of services and the performance of health systems.

**Section 1** focused on integrated care as an approach to serve the population. **Chapter 1** defines the *population-based approach* concept, linking it to major societal transitions, determinants of health and wellness, health services interventions, and healthy aging. In **chapter 2** the authors argue that “quality of health and social services” is a result of the collective work of multiple stakeholders – a population-based perspective. Readers get a deeper insight into the seven dimensions of “quality” and different strategies used to evaluate the quality of health and social services. **Chapter 3** highlights *patient-centered care* – organising health and social services around the needs of clients – as an overarching principle of integrated care. In other words, the population-based approach (the main theme of the book) can be conceived as grounded in patient-centered care. **Chapter 4** discusses collaborative practices – at strategic, tactical and operational levels of the healthcare system – as an essential component of integrated care. **Chapter 5** explores generic forms of integration. The authors discuss the logic underpinning three main typologies of integration: i) horizontal vs vertical integration, ii) dimensions of integration and iii) degrees of integration. **Chapter 6** builds on the previous chapters to present how the bedrock principles of integrated care are connected and operationalised through an in-depth description of eight essential components of an integrated care model. The eighth component – *dedicated coordination of services* – was the main theme of this book’s second section.

**Section 2** focused on exploring coordination of health and social services from a population-based perspective. **Chapter 7** introduces coordination of services by health and social services professionals. Readers get to understand the difference between *ordinary coordination* (carried out by ALL professionals) and *dedicated coordination* (a role for specific professionals). **Chapter 8** explores various forms of dedicated professional coordination of services. This is followed by an introduction to case management – a form of dedicated coordination. **Chapter 9** discusses the roles, duties and competences of case managers. **Chapter 10** highlights the complex role of case mangers. Complex ethical issues faced by case mangers are presented. **Chapter 11** explores conditions for a successful case management program.

Overall, this book is well-written and easy to read. The central theme of the book gives a sense of continuity to readers. Different parts of the narrative are easily connected to the *population-based approach*; this makes for a pleasant reading experience and the message the authors are trying to pass is well understood by readers. They also use information boxes, tables, illustrations and cases studies that highlight their key points – this synthesizes the main ideas of each chapter for the reader. A wide audience may find value in the book. It is rich in theories and principles underlying integrated care policies, clinical and managerial practices – with several references to seminal articles. This is valuable for students who are interested in an in-depth understanding of the fundamental principles of integrated care and their applications. Furthermore, policymakers will be interested in discussions on systemic issues and how they relate to the integrated care philosophy. The authors regularly discuss collaborative practices, tools and strategies in case studies; this is valuable to managers and clinicians who are interested in understanding how integrated care principles can be translated and operationalised in their routine practices. The authors also point out several knowledge translation gaps, advocating for more research in areas such as sustained support and change management strategies in the implementation and routinization of integrated care. Finally, the quality of this book is manifest by its top 5 nomination for the 2016 Donner Prize that rewards the best public policy books written by a Canadian. To the best of my knowledge, this is the only comprehensive book on integrated health and social services written in the French language.

The main weakness of this book is the scope of applications of integrated care principles. The authors regularly referred to Quebec-related policies and practices. It could have been enrichened by including examples from other international contexts. It should be noted that the authors stated in the introduction that they were going to focus on Quebec examples since they were more familiar with their local context. Furthermore, the authors focused on applications related to integrating services for older adults. It can be argued that the population-based approach can also be applicable to other sub-populations who need integrated services like mental health, cancer, children and young people, or maternity care. In my opinion, this book begs to be translated in English in order to reach a wider audience of non-French readers. This book is highly recommended for students and professionals who are interested in understanding the theoretical foundations and practices of integrated care.

